# Anticancer potential of *Thevetia peruviana* fruit methanolic extract

**DOI:** 10.1186/s12906-017-1727-y

**Published:** 2017-05-02

**Authors:** Alberto Ramos-Silva, Faviola Tavares-Carreón, Mario Figueroa, Susana De la Torre-Zavala, Argel Gastelum-Arellanez, Aída Rodríguez-García, Luis J. Galán-Wong, Hamlet Avilés-Arnaut

**Affiliations:** 1 Instituto de Biotecnología, Facultad de Ciencias Biológicas, Universidad Autónoma de Nuevo León, Avenida Universidad S/N, Ciudad Universitaria, CP 66455 San Nicolás de los Garza, NL Mexico; 2 Facultad de Química, Departamento de Farmacia, Universidad Nacional Autónoma de México, CP 04510 Ciudad de México, Mexico

**Keywords:** Cytotoxic activity, Anti-proliferative activity, Motility, Apoptosis, Human cancer cells, Flavonoid, Cardiac glycosides

## Abstract

**Background:**

*Thevetia peruviana* (Pers.) K. Schum or *Cascabela peruviana* (L.) Lippold (commonly known as ayoyote, codo de fraile, lucky nut, or yellow oleander), native to Mexico and Central America, is a medicinal plant used traditionally to cure diseases like ulcers, scabies, hemorrhoids and dissolve tumors. The purpose of this study was to evaluate the cytotoxic, antiproliferative and apoptotic activity of methanolic extract of *T. peruviana* fruits on human cancer cell lines.

**Methods:**

The cytotoxic activity of *T. peruviana* methanolic extract was carried out on human breast, colorectal, prostate and lung cancer cell lines and non-tumorigenic control cells (fibroblast and Vero), using the MTT assay. For proliferation and motility, clonogenic and wound-healing assays were performed. Morphological alterations were monitored by trypan blue exclusion, as well as DNA fragmentation and AO/EB double staining was performed to evaluate apoptosis. The extract was separated using flash chromatography, and the resulting fractions were evaluated on colorectal cancer cells for their cytotoxic activity. The active fractions were further analyzed through mass spectrometry.

**Results:**

The *T. peruviana* methanolic extract exhibited cytotoxic activity on four human cancer cell lines: prostate, breast, colorectal and lung, with values of IC_50_ 1.91 ± 0.76, 5.78 ± 2.12, 6.30 ± 4.45 and 12.04 ± 3.43 μg/mL, respectively. The extract caused a significant reduction of cell motility and colony formation on all evaluated cancer cell lines. In addition, morphological examination displayed cell size reduction, membrane blebbing and detachment of cells, compared to non-treated cancer cell lines. The *T. peruviana* extract induced apoptotic cell death, which was confirmed by DNA fragmentation and AO/EB double staining. Fractions 4 and 5 showed the most effective cytotoxic activity and their MS analysis revealed the presence of the secondary metabolites: thevetiaflavone and cardiac glycosides.

**Conclusion:**

*T. peruviana* extract has potential as natural anti-cancer product with critical effects in the proliferation, motility, and adhesion of human breast and colorectal cancer cells, and apoptosis induction in human prostate and lung cancer cell lines, with minimal effects on non-tumorigenic cell lines.

**Electronic supplementary material:**

The online version of this article (doi:10.1186/s12906-017-1727-y) contains supplementary material, which is available to authorized users.

## Background

Cancer is one of the most important causes of death worldwide. In 2012, 8.2 million cancer-related deaths were reported and the number of new cases is expected to rise by about 70% over the next two decades [1]. Cancer is characterized by the uncontrolled and invasive growth of cells. The most peculiar feature of cancer cells is their ability to metastasize to other specific organs. For example, prostate and colon cancer metastasize to bones and liver, respectively; lung cancer cells spread to adrenal glands, liver, brain, and bones, while breast cancer cells metastasize to lungs and bones [2]. For the most important adenocarcinomas, such as lung, breast, or colorectal cancer, the treatment is barely effective, due to metastatic disease responding only transiently to conventional treatments [3]. Therefore, scientists are still utilizing new alternatives in attempts to find novel compounds and strategies for the treatment of this disease [4].

Plants are a valuable source of new biologically active molecules due to the presence of hundreds of biologically active components [5]. One of the plant families i.e., Apocynaceae, is well-known for anticancer activities, mainly in the genera of *Catharanthus*, *Nerium*, *Strophanthus, Apocynum* and *Thevetia* [6]. *Thevetia peruviana* (Pers.) K. Schum, also known as *Cascabela peruviana* (L.) Lippold, yellow oleander or lucky nut in the West Indies, shows a diverse array of properties ranging from being a cardiotonic to a toxin. *T. peruviana* plant has been used traditionally for the treatment of gastrointestinal and inflammatory diseases, heart failures and skin tumors [7, 8]. All parts of the plant are poisonous due to the presence of cardiac toxins, but the fruit of *T. peruviana*, is the most toxic part of the plant because it has the highest and diverse content of cardiac glycosides. There are many known cases of intentional and accidental poisoning of humans through ingestion of fruits and leaves [9]*.* Whereas about 10 fruits consumed may be fatal for an adult, a single fruit may be lethal for a child. The common clinical set of symptoms resembles digitalis poisoning with marked nausea, vomiting, abdominal pain, diarrhea, dysrhythmias, and hyperkalemia [10]. Although the anticancer potential of parts of *T. peruviana* plant, such as leaves, bark and seeds has been evaluated against human gastric and pancreatic cancer cell lines [11], the anticancer potential of *T. peruviana* fruit is still unknown because only a few cardiac glycosides have been identified and examined as cytotoxic agents. Therefore, the *T. peruviana* fruit extract has been investigated in this study to explore its anticancer potential against the most common cancer types (lung, breast, prostate and colorectal), in terms of morphological analysis, motility and cell adhesive properties, DNA damage and induction of apoptosis in human cancer cell lines.

## Methods

A *T. peruviana* plant specimen was collected in San Nicolás de los Garza, N.L., México (25°43′59.57″N, 100°16′4.75″W). Botanical authentication of the material was performed by the Botany Department Staff of Facultad de Ciencias Biológicas, UANL. A specimen of *T. peruviana* was recently deposited in the Herbarium of the Facultad de Ciencias Biológicas, Universidad Autónoma de Nuevo León (voucher UNL-028732).

### Extraction and preparation of plant samples for testing

The plant extracts were obtained from samples (20 g) of dried roots, leaves, or aerial parts through maceration with methanol (3 × 300 mL) at room temperature for 24 h and under continuous shaking. The methanolic extracts were filtered and evaporated under reduced pressure using a rotary evaporator (Yamato RE801). Samples were stored at −20 °C prior to further experiments. For the cytotoxicity assays, the dried methanol extract was dissolved in dimethylsulfoxide (DMSO) in order to obtain a final concentration of 100 mg/mL (stock) and diluted in PBS (phosphate buffer saline). DMSO concentration in the culture medium was less than 0.1%. Doxorubicin (10 μg/mL, Zytokil) treated cells and untreated cells were used as positive control and negative control, respectively. The methanolic extract of *T. peruviana* was fractionated by column chromatography. Briefly, 1.5 g of fruit extract was adsorbed onto Celite 545 and subjected to reverse flash chromatography on a 50 g RediSep Rf Gold HP C_18_ column, eluting with 10:90 MeOH-H_2_O for 4 CV. Then a linear gradient from 10:90 MeOH-H_2_O to 100% MeOH for 25 CV, holding 100% MeOH for 8 CV, at a flow rate of 40 mL/min to give 120 fractions each containing 12 mL. The resulting fractions were then pooled according to their ELSD and UV profiles, which resulted in six combined fractions in total. All fractions were examined by UPLC-PDA-HRMS-MS/MS and tested for cytotoxic activity against human prostate carcinoma cell line (HTB-81) by the MTT colorimetric assay.

### Cell lines and cell culture

The cancer cell lines used in this study were human colorectal adenocarcinoma (HTB-38), lung carcinoma (HTB-177), prostate adenocarcinoma (HTB-81), and breast adenocarcinoma (HTB-22), whereas the normal cell lines used were human skin fibroblast (CCL-116) and Vero cell line (CCL-81). Cell lines were obtained from the American Type Culture Collection (ATCC). Cell lines were cultured in DMEM or RPMI (only for HTB-81) supplemented with 10% (*v*/v) fetal bovine serum (Byproductos). All cells were cultured at 37 °C under a humidified atmosphere containing 5% CO_2_.

### Cytotoxicity assay

For the cytotoxicity assay, 5 × 10^4-^6 × 10^4^ cells for colorectal, lung, breast, prostate cancer cells, and normal cells were seeded in 96-well tissue culture plates. When the cells had reached 75% confluence, they were incubated using the methanol extracts at various concentrations. After 24 h of incubation, the cytotoxicity was assessed using the MTT assay as described previously [12]. To obtain the half maximal inhibitory concentration (IC_50_)_,_ the percentages of cell viability and growth inhibition were calculated according to the following equations [13]. Cell viability (%) = [(OD of treated cells-OD of blank)/ (OD of control-OD of blank)] × 100. Growth inhibition (%) = 100 – Cell viability (%). All determinations were performed five times independently with three technical replicates.

### Clonogenic assay of cell in vitro

The clonogenic assay was carried out as previously described [14]. 100–130 cells/well and incubated for 24 h at 37 °C with 5% CO_2_ to lead attach. The cells were treated with the corresponding IC_50_ for 24 h and were cultured for 7–14 additional days. Then, the cultures were stained with 0.5% crystal violet for 30 min. Colonies containing more than 50 cells (after 10–14 days of incubation) were counted using ImageJ software [15]. Three independent experiments were performed with three technical replicates each one.

### Wound and healing assay

Wound and healing assay was carried out as previously described [16]. 5 × 10^4^–6 × 10^5^ cells were seeded and grown overnight to reach 100% confluency. The monolayer was treated with the corresponding IC_50_ for 24 h. The cells were migrated into the scratched area and photographed (camera infinity 1–2, Lumenera Corp., CA) every 24 h. The migrated cells were expressed as a mean value per field. Three independent experiments were performed with three technical replicates each one.

### Cell morphology and membrane permeability assays

Cells were seeded in a 96-well plate for 24 h. After attachment, the cells were treated with *T. peruviana* fruit extract using the IC_50_ concentration for 4, 8, 16 and 24 h. For cell morphology, the cells were observed using an inverted microscope (Olympus IX71). Trypan blue assay was used for the permeability assay (0.4% trypan blue/per well) [17]. As a negative control, cells were cultivated in the same plate without the plant extract. Each sample was observed under the microscope and photographs were taken immediately after staining. Three independent experiments were performed with three technical replicates.

### DNA fragmentation analysis

DNA fragmentation was carried out as described previously [18]. Cells were grown in the presence or absence of *T. peruviana* fruit extract using the IC_50_ concentration for 24 h. Doxorubicin treated cells were used as positive control. Briefly, 5 × 10^5^ cells were lysed in DNA lysis buffer [1 M Tris-HCl (pH 8.0), 0.5 M EDTA, 100% Triton X-100, 2% SDS, 0.2 M NaCl], and then, DNA was extracted. The nucleic acid concentration and purity were measured using a NanoDrop® ND-2000 spectrophotometer (Thermo Scientific). Equal amounts of DNA (10 μg/well) were electrophoresed in 1% agarose gel. DNA fragments were visualized using an UV transilluminator (MutilDoc-it Digital Imaging System UVP). Three independent experiments were performed.

### Dual acridine orange/ethidium bromide (AO/EB) fluorescent staining

For the AO/EB method, 5 × 10^5^ cells were seeded in a 6-well plate. Cells were treated with *T. peruviana* fruit extract at the corresponding IC_50_ concentration for 4 h. Then cells were subjected to AO/EB staining as described previously [19]. Briefly, cells were tripsinized and re-suspended in cold PBS and AO/EB dye mix (100 μg/mL AO and 100 μg/mL EB; Sigma) was added. Stained cell suspensions (10 μL) were viewed and counted using a Nikon eclipse TS100 inverted microscope at 40× magnification with excitation filter 480/30 nm and barrier filter 535/40 nm. Six fields per sample were examined. The AO/EB staining method was repeated 3 times.

### Liquid chromatography - Mass spectrometry analysis

HRESIMS data were collected and in positive and negative ionization modes using a Thermo QExactive Plus mass spectrometer (ThermoFisher) equipped an electrospray ionization (ESI) source and via an Acquity UPLC system (Waters Corp). The higher-energy collisional dissociation (HCD) cell used a normalized collision energy of 30 eV for all the compounds to obtain MS/MS data. The UPLC separation was performed using an Acquity BEH C_18_ column equilibrated at 40 °C and a flow rate set at 0.3 mL/min. The mobile phase consisted of 15% CH_3_CN–H_2_O (0.1% formic acid) for 0.5 min, and then a linear gradient from 15% CH_3_CN to 100% CH_3_CN over 6 min, and 1 min holding 100% CH_3_CN before returning to the starting conditions. Samples were dissolved in MS grade methanol and filtered through a 0.2 μm Acrodisc (Waters) filter. Tentative metabolite identification was performed by comparison of HRMS data, UV maxima and fragmentation patterns (MS/MS data) with those contained in the Dictionary of Natural Products [20] reference compounds refined for *Thevetia* plant metabolites.

### Data Analysis

Statistical analysis was performed using GraphPad Prism 7 software. The *p*-value was analyzed in comparison to the untreated samples using Student’s *t*-Test or using one-way analysis of variance (ANOVA) followed by a Tukey test for comparison between different treatment groups. Differences were considered statistically significant at *p* < 0.05. Results were expressed as mean ± SEM of data obtained from tripled or quintuplet, independent experiments. Multivariate analysis was performed using the R 3.2.5 environment. Three biological replicates for each cell line were evaluated, and the variables used were the IC_50_, the clonogenic assay (CL), the membrane permeability (MP) and the wound-healing assay (WH). A standardized principal component analysis (PCA) was performed with “ade4” package [21], followed by an independent component analysis (ICA) applied to the PCA patterns [22].

## Results

### Cytotoxic activity of *T. peruviana* fruit methanol extract on human prostate, breast, colorectal, and lung cancer cell lines

In order to assess the cytotoxic effect of the *T. peruviana* methanol extract on breast (HTB-22), colorectal (HTB-38), prostate (HTB-81), and lung (HTB-177) cancer cell lines, a MTT assay was performed. IC_50_ values indicate the concentration of the extract that inhibits the growth of 50% of the cell population. The criteria of cytotoxicity established by the U.S. National Cancer Institute (NCI) considers a crude extract as active, moderately active, or inactive, when the IC_50_ values are lower than 20 μg/mL, from 20 to 100 μg/mL, or higher than 100 μg/mL, respectively [23]. *T. peruviana* methanol fruit extract induced strong cytotoxicity in all four cancer cell lines (<20 μg/mL), but prostate cancer cells showed the lowest IC_50_ after treatment with *T. peruviana* extract (IC_50_, 1.91 ± 0.76 μg/mL; Table 1). In contrast, the methanol extract of *T. peruviana* exhibited moderate activity against Vero cells and it was inactive against fibroblast cell line (Table 1). Accordingly, the *T. peruviana* fruit extract is cytotoxic to human cancer cell lines, but moderately active on Vero cells and inactive on healthy human fibroblast cells.

**Table 1:** IC_50_ values of *T. peruviana* fruit extract on prostate, lung, colorectal and breast cancer cell lines

IC_50_ (μg/mL) ± SEM					
Cancer cell lines				Normal cell lines	
Prostate	Breast	Colorectal	Lung	Vero	Fibroblast
1.91 ± 0.76^a^	5.78 ± 2.12^a^	6.30 ± 4.45^a^	12.04 ± 3.43^a^	57.02 ± 14.8^b^	1578 ± 301^c^

Human cancer cell lines were treated with different concentrations of *T. peruviana* fruit extract in 96-well microcultured plates for 24 h. IC_50_ values are expressed as mean ± standard error of mean (S.E.M) of quintuplicate determinations. Different letters represent statistically significant differences determined by one way ANOVA (*ρ* < 0.05), followed by Tukey’s multiple comparison test

### *T. peruviana* fruit methanol extract inhibits the proliferative ability of four human cancer cell lines

**Fig. 1:**
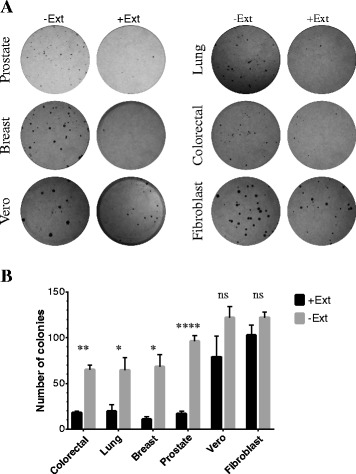
Clonogenic assay of four human cancer cell lines treated with methanol *T. peruviana* extract. **a** Image showing colonies produced by human cancer lines following plating of 100 cells and 10–14 days incubation. Cells were treated with IC_50_ value corresponding for each cell line; **b** cells were quantified and error bar indicates mean ± SEM of three independent experiments. The level of significance was determined using Student’s *t*-Test with ^ns^representing *ρ* > 0.05, **** indicates *ρ* < 0.0001, ** indicates *ρ* < 0.01 and * indicates *ρ* < 0.05

The effect of the *T. peruviana* extract on colony forming ability in four cancer cell lines was investigated. Clonogenic assay was carried out to assess the differences in reproductive viability between treated and untreated cells with *T. peruviana* extract. One hundred cancer cells were seeded in the presence or absence of *T. peruviana* extract, and after 10–15 days of incubation, the colonies formed were counted. The number of colonies observed was significantly inhibited by the *T. peruviana* extract (Fig. 1a). Colony forming ability was reduced by 80% in the breast and prostate cancer cells, while colorectal and lung cancer cells were reduced by 70% (Fig. 1b). In contrast, no defect in proliferation ability was observed for normal cell lines (Fig. 1a, b). Positive control (doxorubicin) inhibited the colony forming ability of both cancer and normal cell lines (Additional file 1: Figure S1). These results suggest that the *T. peruviana* fruit extract has strong anti-proliferative activity against different types of cancer cells without displaying alterations on normal cell lines.

### *T. peruviana* fruit extract affects the cell motility of human cancer cell lines

The effect of the *T. peruviana* fruit extract on cell motility was researched using a wound-healing assay. Cancer cells were evaluated in the presence or absence of the plant extract, monitoring until the induced wound was completely closed. Untreated cells exhibited complete wound closure at 24, 48, 96, and 144 h for the human prostate, breast, lung, and colorectal cancer cell lines, respectively. In contrast, all cancer cell lines evaluated showed a dramatic inhibition of wound closure when treated with the *T. peruviana* extract. The wound closure of Vero and fibroblast cells was achieved at 48 h, whether exposed to the fruit extract or not (Additional file 2: Figure S2). As a positive control, doxorubicin was used in wound-healing assay and showed an increase in the wound due to strong detachment of both the human cancer and normal cell lines (Additional file 3: Figure S3). These results indicated that the *T. peruviana* fruit extract inhibited and delayed the migration of four different human cancer cells *in vitro*.

### Morphological changes in four human cancer cell lines exposed to *T. peruviana* extract

**Fig. 2:**
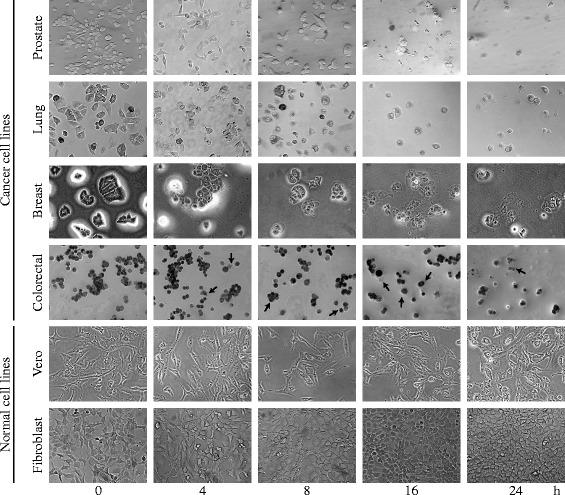
Morphological changes on four human cancer cell lines during treatment with *T. peruviana* methanol extract. Human cancer cells were treated at IC_50_ corresponding value for each cell line and monitored over a period of 24 h. *Black arrows* indicated blebbing membranes

All the human cell lines displayed significant morphological changes when treated with *T. peruviana* fruit extract for 24 h (Fig. 2). Thus, we checked whether changes in cell morphology occurred during the incubation of the cells with media containing plant extract. Morphological changes of human cancer cells treated with plant extract were monitored up to 24 h. At 8 h of incubation, some cells were rounded up in the prostate, lung, and breast cancer cells. After 16 h of treatment, the cells had shrunk and showed disrupted intercellular contacts. In some cases, the cells were bi-nucleated and the majority of the cells were detached from the wells, denoting cell death. These changes were more drastic for the prostate, breast, and lung cancer cell lines than for colorectal cells and the most prominent effect in the latter was membrane blebbing (small protrusions of the membrane). Because cells did not show vacuolation in the cytoplasm, we considered that autophagy is not involved in the mechanism of cell death [24]. To highlight, normal cell lines showed no evident morphology alteration during the 24 h of treatment with *T. peruviana* fruit extract. When control treatment with doxorubicin was applied, cell morphology was altered in both human cancer and normal cells (Additional file 4: Figure S4). These results indicated that after 8 h of exposure to the *T. peruviana* extract, the cancer cells presented significant morphological alterations without any observed change in the healthy cell lines.

### *T. peruviana* extract affects the viability of human cancer cells in a time-dependent manner

**Fig. 3:**
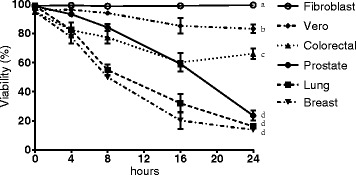
Viability of four human cancer cells exposed to *T. peruviana* methanol extract over a period of 24 h. Human cancer cells were treated at IC_50_ value corresponding for each cell line. Cell viability was evaluated using the trypan blue exclusion method. Each data point represents values from three independent experiments (*n* = 3). Error bar indicates mean ± SEM. Different letters represent statistically significant differences determined by one way ANOVA (*ρ* < 0.05)

To determine whether the viability of the human cancer cells is affected during treatment with *T. peruviana* extract, the trypan blue exclusion test was used. Loss of the integrity of plasma membrane can be verified using polar dyes, such as trypan blue, which is excluded by an intact membrane. We observed that the number of dead cells increased in a time-dependent manner in the presence of the extract plant. The effect of the *T. peruviana* extract on cell viability of the breast and prostate cancer cells was 50–60% viability after 8 h of treatment, whereas the lung and colorectal cancer cells had 80% viability after 8 h. After 24 h, the lung, prostate, and breast cancer cells showed viability decreased to below 25% (Fig. 3). Interestingly, the colorectal cancer cells were less sensitive to plant extract showing 70% of viability after 24 h treatment. Vero and fibroblast cells showed a small decrement or none at all in viability during the treatment with the *T. peruviana* fruit extract. This result confirms that *T. peruviana* extract has a cytotoxic effect (at the level of membrane integrity) on different types of cancer cells whereas the normal fibroblast cells were not affected.

### Motility and membrane permeability features are mainly affected by *T. peruviana* fruit extract

**Fig. 4:**
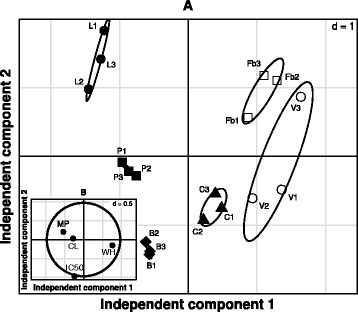
Classification of normal and cancer cell lines exposed to *T. peruviana* fruit methanolic extract according to independent component analysis (ICA). The distribution of the cell lines (panel A, projections with 95% confidence ellipses) and variables (panel B, projection of variable loadings, with maximum loading indicated by a circle) is shown in the space spanned by the two leading independent components. The clear unsupervised discrimination among the six cell lines reflects the greater effect of *T. peruviana* extract on tumor cell lines (lung cells, L, ●, prostate cells, P, ■, breast cells, B, ♦, colorectal cells, C, ▲), while normal cells are less affected (fibroblast cells, Fb, □, Vero cells, V, ○). The independent component 1 is clearly separating cancer cells from normal cells, mainly due to the effect of the extract on the motility (WH) and membrane permeability (MP), while the independent component 2 is separating samples mainly by the IC_50_ value observed for each cell line

Multivariate analysis was performed on the six cell lines (four tumoral and two normal), using as variables to be evaluated: the IC_50_ value, the clonogenic assay, the wound healing assay, and membrane permeability. PCA was applied to the whole dataset and the first 3 principal components (PC’s) accounted for 97.3% of the observed variability. Assuming that directions with the greatest variance are the most biologically relevant, these leading PC’s were selected as those with the major structure or variance, as a de-noising step enhancing ICA results. ICA obtained 3 independent components (IC1, IC2 and IC3) with negative kurtosis (−1.62, −1.42 and −0.12, respectively). The employment of these components, plotting IC1 and IC2, allowed the effective separation of samples into their original groups (Fig. 4). With this, ICA was able to distinguish between the sensitivity of normal (right side) and cancer cells (left side) exposed to *T. peruviana* extract (with colorectal cancer cell between both populations). The IC1 clearly separates tumor cells from normal cells, mainly due to the effect of the extract on cell motility (wound healing assay) and the membrane permeability, while the IC2 separates samples mainly on the basis of the IC_50_ observed for each cell line. The anti-proliferative (clonogenic assay) response of the cell lines to the extract is mainly represented by the IC3, which can be observed in the Additional file 5: Figure S5. Overall, this analysis identified that the main features affected by the *T. peruviana* extract were cell motility and membrane permeability and, less significantly, the IC_50_ and anti-proliferative activity.

### *T. peruviana* fruit extract induces DNA fragmentation in four types of human cancer cells

**Fig. 5:**
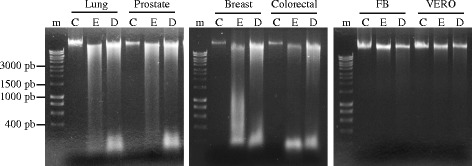
DNA fragmentation of human cancer cell lines treated with *T. peruviana* methanol extract. Lung, prostate, breast and colorectal cancer cell lines were treated with the corresponding IC_50_ value for each cell line during a 24 h period; m, molecular-weight marker; C, untreated control; E, cancer cell line treated with *T. peruviana* methanol extract at its corresponding IC_50_ value and, D, cancer cell line treated with doxorubicin (10 μg/mL). Representative result from three independent experiments is shown

The detection of the DNA fragmentation is a common hallmark of cells undergoing late-stage apoptosis [25]. In order to determine if *T. peruviana* fruit extract could induce DNA fragmentation and thus whether apoptosis occurred, human cancer cells exposed to *T. peruviana* treatment were assessed for DNA laddering and visualized by agarose gel electrophoresis (Fig 5). It was found that the four human cancer cell lines incubated with *T. peruviana* extract showed apoptotic DNA fragmentation profiles similar to the positive control, doxorubicin, which is known to induces apoptosis [26]. No nucleic acid fragmentation was observed in untreated cells. Normal cell lines showed a lower degree of DNA fragmentation compared with cancer cell lines.

### Apoptosis induction by *T. peruviana* fruit extract on human lung and prostate cancer cells

**Fig. 6:**
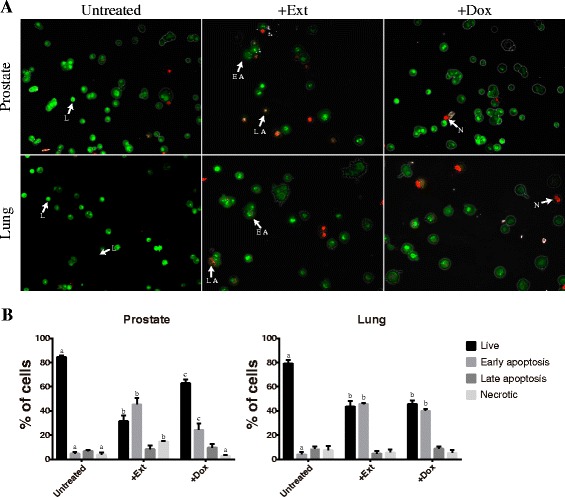
AO/EB double stain of human cancer cell lines after a treatment with *T. peruviana* methanol extract. **a**. Prostate and lung cells were treated with IC_50_ of *T. peruviana* methanol extract. Images represent the control (untreated cells), treated cells and, cell treated with doxorubicin (10 μg/mL) as positive control. Cells were stained with acridine orange and ethidium bromide (AO/EB) after 4 h of treatment. White arrows indicate live (L), early apoptotic (EA), late apoptotic (LA) or necrotic (N) cells. **b**. Error bar indicates mean ± SEM of three independent experiments. +Ext and +Dox, both indicate cells treated with extract or doxorubicin. Different letters represent statistically significant differences determined by one way ANOVA (*ρ* < 0.05) between bars with same color by cell line. Non-letter bars have no statistical difference

It was observed that *T. peruviana* extract triggered morphological changes and DNA fragmentation in a time-dependent manner that could be related to apoptosis. To determine and quantify cell death, acridine orange/ethidium bromide (AO/EB) fluorescent staining was used to identify apoptosis-associated changes in the cell membrane. AO is a membrane-permeable dye that binds to the nucleic acids of viable cells. EB is an impermeable dye, but readily penetrates the membrane of nonviable cells and binds to DNA. When AO/EB are used simultaneously, viable cells fluoresce green and nonviable cells fluoresce red under fluorescence microscopy. Lung and prostate cells were labeled by AO/EB after 4 h of treatment with the *T. peruviana* extract, and dual staining was examined under a fluorescent microscope. Early and late-stage apoptotic cells, marked by crescent-shaped or granular yellow-green AO nuclear staining (as indicated by condensed or fragmented chromatin) were detected in the treated group (with *T. peruviana* extract) and the positive control (doxorubicin treated) (Fig. 6a). Also, dead cells from direct necrosis were detected, having an orange nucleus. No significant apoptosis was detected in the negative control group (untreated cells), which showed a normal green nucleus. Quantification of the live, apoptotic, and necrotic cell populations in the control and treated human prostate and lung cancer cells indicated that after 4 h of treatment, the prostate and lung cancer cells increased the early apoptosis stage by 40.65% and 41.51%, respectively (Fig. 6b). Thus, in accordance with our DNA fragmentation results, the *T. peruviana* fruit extract triggers apoptosis.

### Metabolic profiling of *T. peruviana* fractions by LC-MS analysis

Methanol extract of *T. peruviana* fruit was subjected to flash chromatography and six fractions were obtained. Because prostate cancer cells showed the lowest IC_50_ to *T. peruviana* crude extract compared to lung, breast, and colorectal cancer cells, every single fraction was analyzed on the prostate cancer cell line by MTT assay, fractions four and five being the most significantly cytotoxic (Table 2). Those fractions were analyzed using UPLC-PDA coupled with a HRESIMS-MS/MS spectrometer. Each peak contained in the fractions was dereplicated for their UV profile, HRMS data in both positive and negative modes, and for its MS/MS fragmentation pattern against a refined subset of plant metabolites of *Thevetia* species contained in the Dictionary of Natural Products (286,310 compounds contained in the database). The results showed that these fractions contained the polyketide thevetiaflavone and the cardiac glycosides: peruvosidic acid, peruvoside, thevefoline, solanoside, neriifoside and neriifolin.

**Table 2:** Cell viability of the fractions of *T. peruviana* fruit extract on prostate cancer cell line (HTB-81)

Fraction	% Viability
1	116 ± 3.7^a^
2	121 ± 5^a^
3	103 ± 4^a^
4	3.71 ± 3.4^b^
5	1.24 ± 1.9^b^
6	62 ± 3^c^

The fractions were obtained by flash chromatography. Viability was determined by MTT assay, values being expressed as mean ± SEM of triplicate determinations. Different letters represent statistically significant differences determined by one way ANOVA (*ρ* < 0.05), followed by Tukey’s multiple comparison test

## Discussion

*T. peruviana* belongs to the Apocynaceae family, a plant which is native to central and southern Mexico, as well as Central America. It is a medicinal plant used to treat different diseases, including cancer [27]. The leaves of yellow oleander had previously been studied and shown to possess antimicrobial, antifungal, antidiarrheal, insecticide, molluscacide, and rodenticide activity [8, 28–30]. However, scientific evidence to demonstrate mode of action, targets and agents responsible for the bioactivity in the fruit is still needed. The purpose of the present study was to find out the cytotoxic and anti-proliferative activity of methanol extracted fruit of *T. peruviana* on different types of human cancer cell lines.

Our experiments in vitro showed that *T. peruviana* fruit extract exhibited strong cytotoxicity against four cancer cell lines. Among the cell lines examined, lung cells showed higher IC_50_ value (12.04 μg/mL) than prostate (1.91 μg/mL), breast (5.78 μg/mL), and colorectal (6.30 μg/mL) cells. Such variation among the cell lines might be attributed in part to the fact that cancer cells possess differences in their genetic make up, morphology and doubling time, resulting in differential susceptibility to the same cytotoxic agent [31]. Our results, together with previously reported toxic activity, suggest that *T. peruviana* fruit methanol extract has an antiproliferative potential.

Cancer is a complex disease characterized by proliferation of highly resistant cells to death. An increased rate of cellular proliferation is frequent, due to the fact that most cancer cells divide more often than normal cells. The goal of targeting cell proliferation is to arrest the cell cycle or induce cancer cell death using cytotoxic compounds. We show here, that *T. peruviana* fruit extract significantly reduced the cell viability and the ability of cells to form colonies in four different human cancer cell lines. The clonogenic assay has been used to detect cells that have retained their capacity to produce a large number of progeny after radiation and chemotherapy treatments [32]. It also correlates tumorigenicity analysis in vivo and predicts the clinical response toward several agents in breast cancer patients [33]. Hence, our results in clonogenic assay suggest that *T. peruviana* fruit extract has potential anticancer activity, limiting proliferation of cancer cells after treatment. Notably, normal cells (vero and fibroblast) treated with *T. peruviana* extract did not show any observable effect in clonogenic assay.

Migration is a critical step in initial progression of cancer that facilitates metastasis. Wound and healing assay is a classic and common method used for discovery and validation of molecules that affect cell migration [16, 34] and metastasis [35]. The methanolic *T. peruviana* fruit extract inhibited and delayed the cell migration of cancer cells, but not among normal cells. These results open the door to further studies that could confirm if the cytotoxic activity of *T. peruviana* fruit extract alters the regulation of the actin cytoskeleton, induces morphological changes and leads to detachment of cells, culminating in cell death. Moreover, motility and membrane permeability features were mainly affected by *T. peruviana* fruit extract, according with the multivariate analysis performed on the six cell lines and the four variables evaluated, suggesting a potential antimetastatic activity in the *T. peruviana* fruit extract.

One crucial and desirable mechanism by which chemotherapeutics destroy tumor cells is by inducing apoptosis. Cells undergoing apoptosis show morphological and biochemical modifications including chromatin segregation, nuclear condensation, DNA fragmentation, partition of the membrane, and vesicles formation [36, 37]. The late-stage of apoptosis can be visualized by standard agarose gel electrophoresis as a ladder pattern due to DNA cleavage [38], while both (the early and late stages of apoptosis) can be determined by AO/EB fluorescent staining. *Cerbera manghas*, a plant belonging to the Apocynaceae family, contains a cardiac glycoside (neriifolin), which induced DNA fragmentation on hepatocellular carcinoma 48 h after treatment [39]. Here, crude extract from *T. peruviana* fruit showed death induction on cancer cell lines through early apoptosis mechanisms (AO/EB fluorescent staining) 4 h after treatment and late-stage apoptosis (DNA laddering assay) after 24 h of treatment. This result indicates that *T. peruviana* could have a higher level of apoptotic activity than other members of the Apocynaceae family. In addition, it will be important to determine whether the apoptotic activity is located in the compounds present in the flesh or seeds.

Mass spectrometry analysis of active fractions from *T. peruviana* fruit methanol extract indicated that one flavonoid and cardiac glycosides are secondary metabolites present in the fruit plant. Flavonoids, such as curcumin, quercentin and genistein, are known to have cell line-specific anti-proliferative and apoptosis inducing activity [40–42]. It has been postulated that flavonoids possess anticancer properties manifested through several mechanisms, including decrease of reactive oxygen species, inhibition of DNA topoisomerase and downward regulation pathway of nuclear transcription factors [43–46]. Further experiments are required to determine the cytotoxic effect of thevetiaflavone and individual cardiac glycosides present in the methanolic fruit extract of *T. peruviana* on human cancer cell lines. Recent investigations of seeds from *T. peruviana* resulted in the isolation of cardiac glycosides that had inhibitory effects against human gastric and pancreatic cancer cell lines [11]. Cardiac glycosides are the most researched secondary metabolites in *T. peruviana* due to the fact they can heal heart pathologies [47], but are also being studied for their cytotoxic and/or apoptotic activities against myeloid leukemia (peruvoside) [48] and hepatocellular carcinoma (neriifolin) [39]. Thevetin and peruvoside are cardiac glycosides which are clinically important constituents due to be used in treatment of arrhythmias [49]. One hundred nine cardenolides have been isolated and identified from members of the Apocynaceae family, and about a quarter of them are reported to have anticancer activity. Cardenolides are well known as the substrates of Na+/K + −ATPase, which is an enzyme that regulate various cell survival and death signal pathways [6] and its relative distribution and expression are distinct in cancer cells compared with normal ones, indicating that they could serve as a novel target with great potential. This could be the reason that multivariate analysis showed an adequate separation between normal and cancer cells, revealing significant differences in their sensitivity to the toxic compounds in the *T. peruviana* fruit methanolic extract, mainly due to the effect of the extract on the motility and membrane permeability on human prostate, breast, colorectal and lung cancer cell lines. The cytotoxic activity of crude extract from *T. peruviana* fruit could be attributed to their phytochemical components such as thevetin A, thevetin B, peruvoside, thevenerin, and cerberin, which are toxins [50], and also to the cardiac glycosides found in active fractions reported in this study (thevefoline, solanoside, neriifoside, peruvoside and neriifolin). Finally, the isolation of the active principles of the methanolic extract of *T. peruviana* fruit is currently being undertaken to investigate their cytotoxic, molecular and genetic action mechanisms, which could provide meaningful perspectives for biomedical and biotechnological research.

## Conclusions

The current study presents evidence that the methanol extract of *T. peruviana* fruit inhibits cell proliferation, has a time-dependent cytotoxic activity and induces apoptosis of human cancer cell lines, but has minimal or less pronounced effects on normal cells. The fruit extract displayed anticancer properties mainly through mechanisms that included membrane permeability, motility and DNA fragmentation. Maximum cytotoxic activity was observed in a fraction that contained one flavonoid and cardiac glycosides. Chemical analyses of the active fractions are currently in progress to perform a better evaluation of their biological significance. Additionally, further “in vivo” research is essential to show the full potential for the use of *T. peruviana* fruit extract in cancer therapy. In conclusion, these findings shows the importance of *T. peruviana* fruit as a source of bioactive compounds with anticancer potential.

## Additional files

Additional file 1: Figure S1. Clonogenic assay of four human cancer cell lines treated with doxorubicin. Image showing colonies produced by human cancer lines and normal cell lines, following plating of 100 cells and 10–14 days incubation. Cells were treated with doxorubicin at 10 μg/mL. (PDF 1719 kb)

Additional file 2: Figure S2. Wound and healing closure activity of four human cancer cell lines treated with methanol *T. peruviana* extract. Representative photographs of wounded cancer cells monolayer after 24 h of treatment with methanol *T. peruviana* fruit extract at IC_50_ value corresponding to each cell line. Vero and fibroblast cells were used as normal cell lines. A typical result from three independent experiments is shown. (PDF 2.74 mb)

Additional file 3: Figure S3. Wound and healing closure activity of four human cancer cell lines treated with doxorubicin. Representative photographs of wounded cancer cells monolayer after 24 h of treatment with doxorubicin at 10 µg/mL. The result from three independent experiments is shown. (PDF 2.62 mb)

Additional file 4: Figure S4. Morphological changes on four human cancer cell lines during treatment with doxorubicin. Human cancer cells were treated with doxorubicin at 10 µg/mL and monitored over a 24 h period. (PDF 1.88 mb)

Additional file 5: **Figure S5.** Classification of normal and cancer cell lines exposed to *T. peruviana* fruit methanolic extract according to independent component analysis (ICA). The distribution of the cell lines (panel A, projections with 95% confidence ellipses) and variables (panel B, projection of variable loadings with maximum loading indicated by a circle) is shown in the space spanned by the independent components 1 and 3. The clear unsupervised discrimination among the six cell lines reflects the greater effect of *T. peruviana* extract on tumor cell lines (lung cells, L, ?, prostate cells, P, ¦, breast cells, B, ?, colorectal cells, C, ?), while normal cells are affected less (fibroblast cells, Fb, ?, Vero cells, V, ?). The independent component 1 is clearly separating cancer cells from normal cells, mainly due to the effect of the extract on the motility (WH) and membrane permeability (MP), while the independent component 3 is separating samples mainly by the anti-proliferative (clonogenic assay, CL) observed for each cell line. (PDF 1121 kb)

## Abbreviations

AO: Acridine orange
ATCC: American Type Culture Collection
DMEM: Dulbecco’s Modified Eagle’s medium
DMSO: Dimethyl sulfoxide
EB: Ethidium bromide
IC: Independent component
IC_50_: Half maximal inhibitory concentration
ICA: Independent component analysis
MTT: 3-(4,5-dimethylthiazol-2-yl)-2,5-diphenyltetrazolium bromide
PBS: Phosphate buffer saline
PC: Principal component
PCA: Principal component analysis
RPMI: Roswell Park Memorial Institute medium
