# Supplementary figures and images for: Anticancer potential of Thevetia peruviana fruit methanolic extract
Source: BMC Complement Altern Med. 2017 May 2;17:241. doi: 10.1186/s12906-017-1727-y (PMC5414213; doi:10.1186/s12906-017-1727-y)

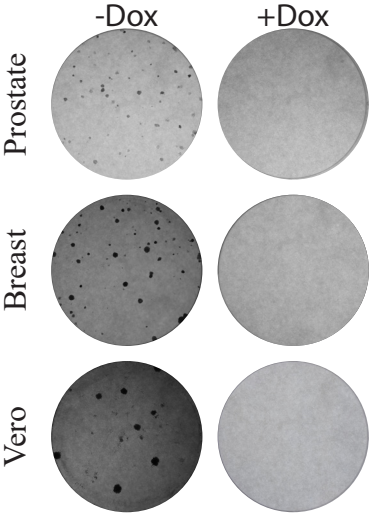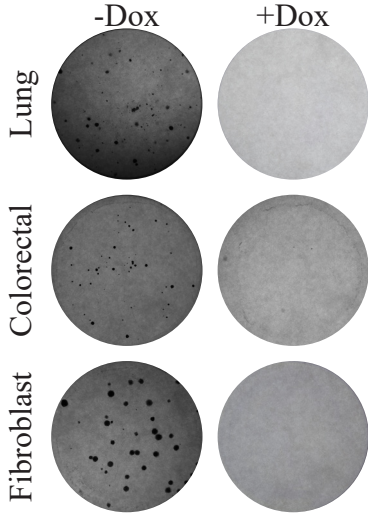

Supplement: Supplementary file 1 — Clonogenic assay of four human cancer cell lines treated with doxorubicin. Image showing colonies produced by human cancer lines and normal cell lines, following plating of 100 cells and 10–14 days incubation. Cells were treated with doxorubicin at 10 μg/mL. (PDF 1719 kb) [file 12906_2017_1727_MOESM1_ESM.pdf]

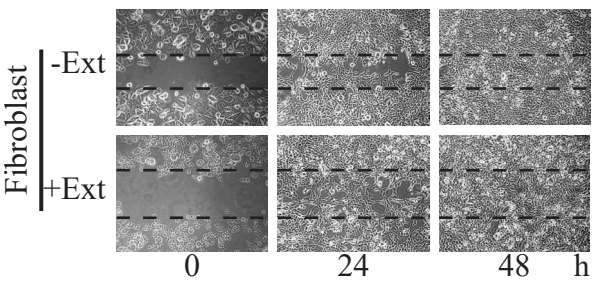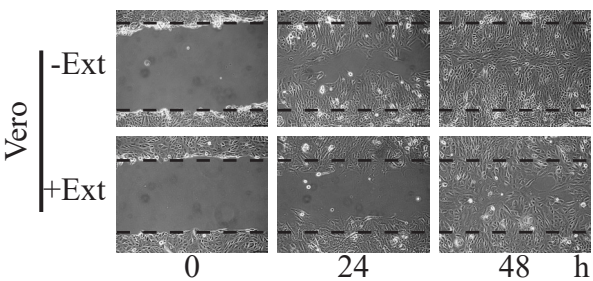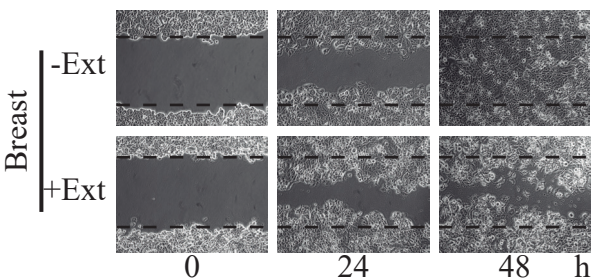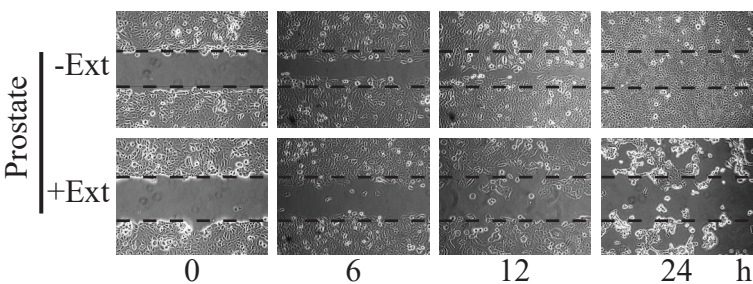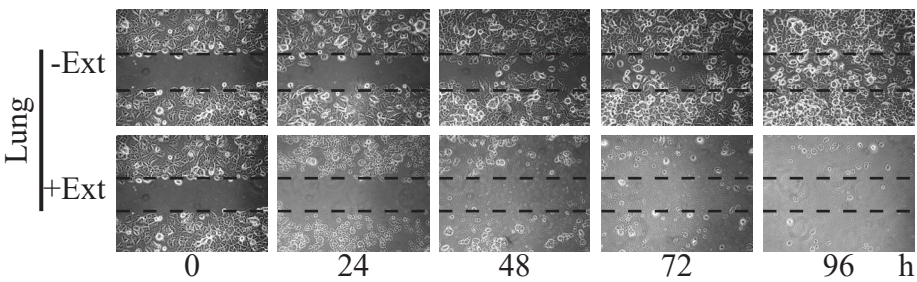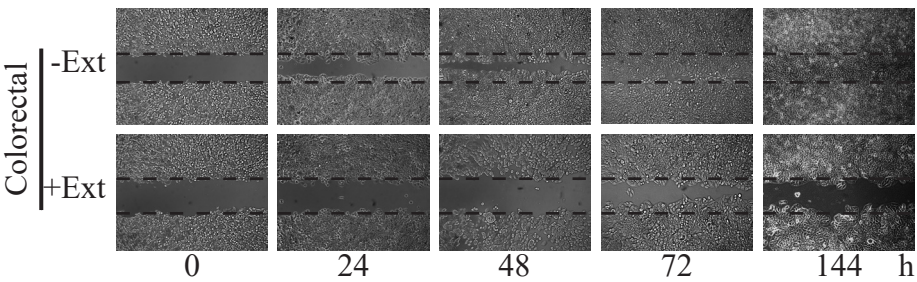

Supplement: Supplementary file 2 — Wound and healing closure activity of four human cancer cell lines treated with methanol T. peruviana extract. Representative photographs of wounded cancer cells monolayer after 24 h of treatment with methanol T. peruviana fruit extract at IC50 value corresponding to each cell line. Vero and fibroblast cells were used as normal cell lines. A typical result from three independent experiments is shown. (PDF 2.74 mb) [file 12906_2017_1727_MOESM2_ESM.pdf]

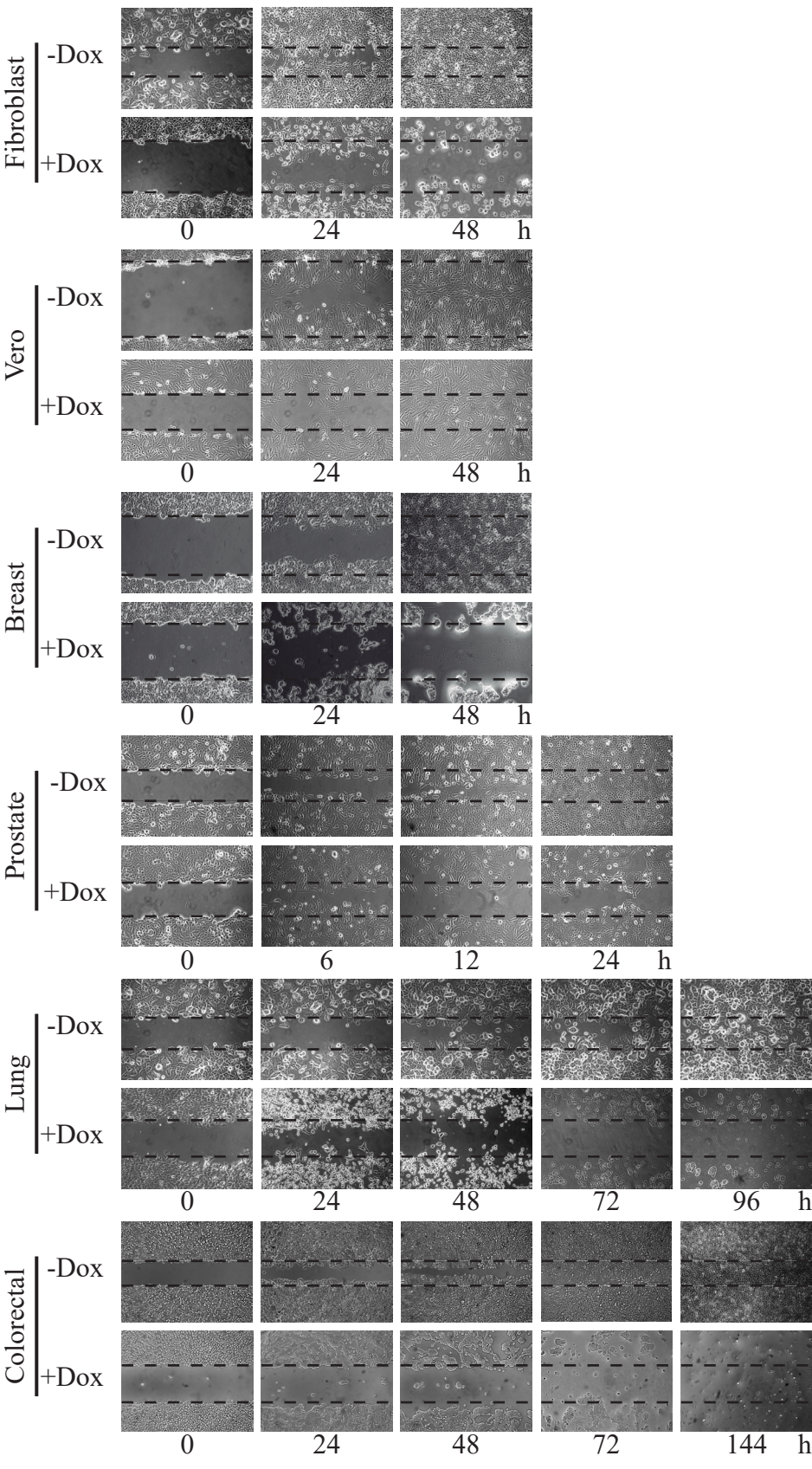

Supplement: Supplementary file 3 — Wound and healing closure activity of four human cancer cell lines treated with doxorubicin. Representative photographs of wounded cancer cells monolayer after 24 h of treatment with doxorubicin at 10 µg/mL. The result from three independent experiments is shown. (PDF 2.62 mb) [file 12906_2017_1727_MOESM3_ESM.pdf]

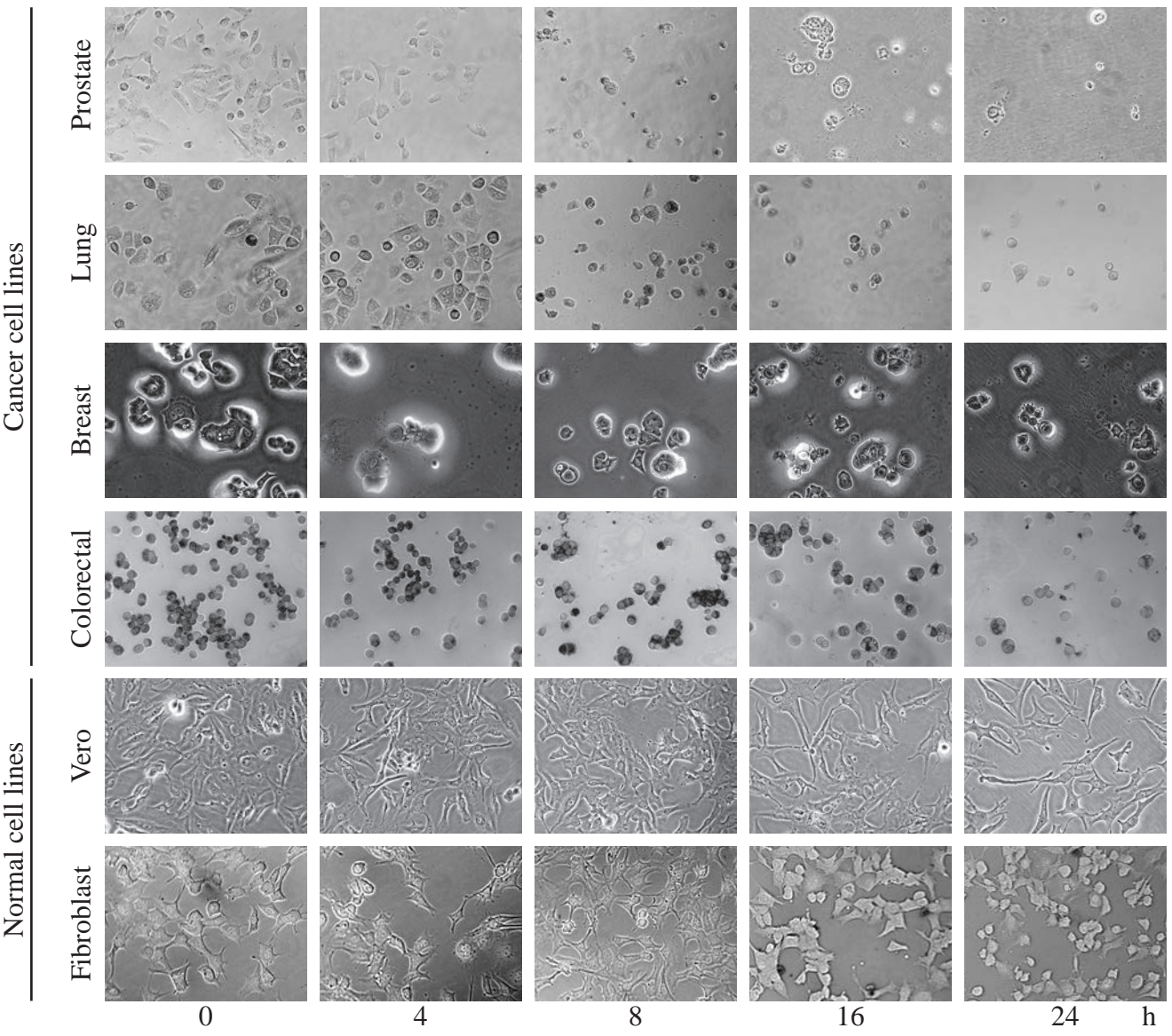

Supplement: Supplementary file 4 — Morphological changes on four human cancer cell lines during treatment with doxorubicin. Human cancer cells were treated with doxorubicin at 10 µg/mL and monitored over a 24 h period. (PDF 1.88 mb) [file 12906_2017_1727_MOESM4_ESM.pdf]
