# Supplementary material for: Anticancer potential of Thevetia peruviana fruit methanolic extract
Source: BMC Complement Altern Med. 2017 May 2;17:241. doi: 10.1186/s12906-017-1727-y (PMC5414213; doi:10.1186/s12906-017-1727-y)
Supplement: Supplementary file 5 — Figure S5. Classification of normal and cancer cell lines exposed to T. peruviana fruit methanolic extract according to independent component analysis (ICA). The distribution of the cell lines (panel A, projections with 95% confidence ellipses) and variables (panel B, projection of variable loadings with maximum loading indicated by a circle) is shown in the space spanned by the independent components 1 and 3. The clear unsupervised discrimination among the six cell lines reflects the greater effect of T. peruviana extract on tumor cell lines (lung cells, L, ?, prostate cells, P, ¦, breast cells, B, ?, colorectal cells, C, ?), while normal cells are affected less (fibroblast cells, Fb, ?, Vero cells, V, ?). The independent component 1 is clearly separating cancer cells from normal cells, mainly due to the effect of the extract on the motility (WH) and membrane permeability (MP), while the independent component 3 is separating samples mainly by the anti-proliferative (clonogenic assay, CL) observed for each cell line. (PDF 1121 kb) [file 12906_2017_1727_MOESM5_ESM.pdf]

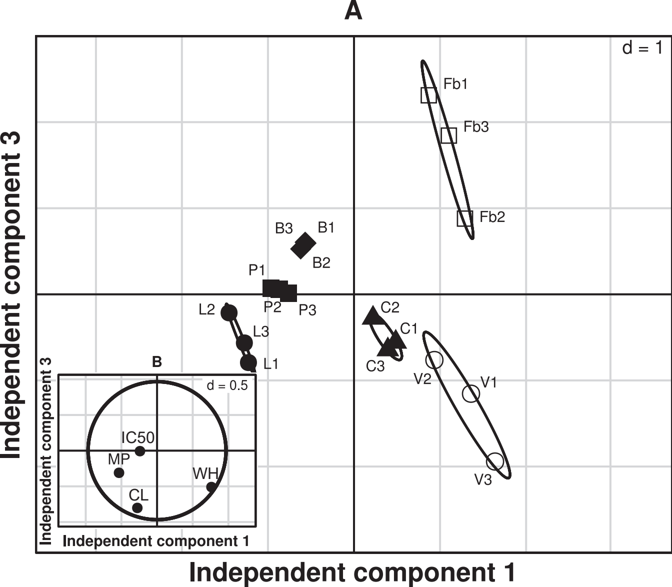
A
d = 1
Fb1
Fb3
Fb2
B3
B1
B2
P1
P2
P3
L2
L3
L1
C2
C1
C3
V2
V1
V3
B
d = 0.5
IC50
MP
WH
CL
Independent component 3
Independent component 1
Independent component 3
Independent component 1
